# Plasma Rich in Growth Factors (PRGF) Versus Saline Intraosseous Infiltrations Combined with Intra-Articular PRGF in Severe Knee Osteoarthritis: A Prospective Double-Blind Multicentric Randomized Controlled Trial with 1-Year Follow-Up

**DOI:** 10.3390/jcm14228075

**Published:** 2025-11-14

**Authors:** Mónica Sánchez Santiuste, Víctor Vaquerizo García, José Antonio Pareja Esteban, Roberto Prado, Sabino Padilla, Eduardo Anitua

**Affiliations:** 1 Department of Orthopaedic Surgery, Príncipe de Asturias University Hospital, 28805 Alcalá de Henares, Spain; vaquerizovictor@yahoo.es (V.V.G.); joseantonio.pareja@salud.madrid.org (J.A.P.E.); 2 Eduardo Anitua Foundation for Biomedical Research, 01007 Vitoria, Spain; roberto.prado@bti-implant.es (R.P.); sabino.padilla1958@gmail.com (S.P.); eduardo@fundacioneduardoanitua.org (E.A.); 3 Regenerative Medicine Laboratory, BTI-Biotechnology Institute IMASD, 01007 Vitoria, Spain; 4 University Institute for Regenerative Medicine & Oral Implantology—UIRMI (UPV/EHU-Fundación Eduardo Anitua), 01007 Vitoria, Spain

**Keywords:** osteoarthritis, knee, PRGF, platelet-rich plasma, regenerative medicine, randomized controlled trial

## Abstract

**Background/Objectives**: Severe knee osteoarthritis (KOA) is a degenerative disease that significantly affects quality of life (QoL). Although intra-articular (IA) injections of plasma rich in growth factors (PRGF) have proven effective, the subchondral bone plays a crucial role in pathogenesis. The objective of this study was to evaluate the efficacy of intraosseous (IO) PRGF infiltrations in comparison with a saline placebo, followed by the conventional standard treatment of three IA PRGF injections, in enhancing clinical outcomes in patients suffering from severe KOA. **Methods**: A prospective, randomized, double-blind, multicenter clinical trial was conducted. Eighty-six patients with Kellgren–Lawrence grade III-IV KOA were randomly assigned to two groups: one received an IO infiltration of PRGF and the other received an IO saline solution. Both groups subsequently received three IA PRGF injections. Clinical outcomes were assessed using the KOOS and WOMAC scales at baseline and at 3, 6, and 12 months. **Results**: Both groups showed a statistically significant improvement in all KOOS and WOMAC subscales at all follow-up points compared to their baseline values. However, the group that received the IO PRGF infiltration demonstrated significantly greater improvements in nearly all domains of the KOOS and WOMAC scales (pain, symptoms, function, and quality of life) at 3, 6, and 12 months compared to the saline group (*p* < 0.05). No serious adverse events were recorded. **Conclusions**: The combination of intraosseous and intra-articular PRGF infiltrations is a superior therapeutic strategy to the combination of intraosseous saline solution and intra-articular PRGF for treating severe KOA. These findings suggest that treating the subchondral bone directly with PRGF has a significant and clinically relevant therapeutic effect, resulting in greater pain reduction and functional improvement at one-year follow-up.

## 1. Introduction

Knee osteoarthritis (KOA) is a highly prevalent, chronic, low-grade, sterile inflammatory condition affecting 37% of people over 60 years of age [1] with distinct heterogeneous phenotypes [2,3,4,5]. The homeostatic dysregulation of the synovial joint’s tissues leads to a progressively painful and functionally disabled knee joint [6,7,8] due to the crosstalk between the inflammatory microenvironment of two highly innervated and well-vascularized cell tissues—the synovium and the subchondral bone—with the synovial liquid and the articular cartilage [9,10]. Although the primary driver and the triggering tissue that ignites KOA remain undetermined [11,12,13,14], the inflammatory and catabolic signaling amongst these tissues, in a feedback-loop manner, will progressively lead to the dysregulation of multiple stromal and immune cells, ending in the functional and structural failure of the knee joint as an organ [12,15,16], with the dysregulation of homeostasis of the subchondral bone playing an outstanding role [3,9,10].

A novel approach to treating KOA is to use intra-articular (IA) injections of plasma rich in growth factors (PRGF), an autologous concentrate of platelets suspended in plasma that, once activated, becomes a fibrin biomaterial soaked up with growth factors coming from platelets and plasma [17,18]. Myriad pleiotropic proteins including but not limited to IGF1, HGF, TGF-β, VEGF, and SDF-1 have been reported to exert immunomodulatory, trophic and antialgic effects on affected joint tissues [3,12,18]. Accordingly, several clinical studies have described pain reduction and improvement of knee function and quality of life in patients undergoing IA PRGF injections [19,20,21,22,23]. In this line of research, the combination of intraosseous (IO) and IA PRGF infiltrations has emerged as an innovative, safe, and minimally invasive “joint-centric” strategy in the treatment of KOA by targeting as many of the affected tissues as possible at once [3,14,24,25], resulting in significant knee joint pain reduction and functional improvement [24,26,27,28,29].

The purpose of this study was to assess, by using KOOS and WOMAC scores, the contribution of PRGF versus saline placebo intraosseous infiltrations followed by the conventional and active treatment of three intra-articular PRGF injections [24] in ameliorating pain and joint function in patients with Kellgren–Lawrence grades III and IV KOA.

## 2. Materials and Methods

### 2.1. Study Design

This study was designed as a double-blind multicentric randomized prospective clinical trial and was conducted at a public hospital in Madrid and another in Jaén (Spain). The study protocol (Code: EC_01_2017) was approved by the hospital’s Institutional Review Board in accordance with international ethical standards from the revised World Medical Association Declaration of Helsinki amended in 2013 in Brazil [30]. The study was registered prior to its initiation in the EU Clinical Trials Register (EudraCT number: 2017-000623-27, registration date: 20 June 2017) and in the Spanish Register of Trials (REec) (Identifier number: 2017-000623-27, registration date: 11 September 2017). Patients were informed of the risks associated with the study and provided written informed consent. The trial was reported following the Consolidated Standards of Reporting Trials (CONSORT) 2025 guidelines (Supplementary Table S1) [31].

### 2.2. Study Population

Patients were included if they met the following criteria: (1) Age between 40 and 75; (2) Both genders; (3) At least 6 months of moderate to severe pain and symptoms according to the KOOS scale; (4) Grades III-IV knee osteoarthritis on the Kellgren and Lawrence scale according to radiological criteria [32]; (5) BMI under 35 Kg/m^2^; (6) No response to other pharmacological treatments; and (7) Able to fulfill the schedule of trial visits and maintain adequate follow-up.

Patients were excluded from the study if any of the following characteristics were presented: (1) Intra-articular infiltration with PRGF in the 12 months before inclusion; (2) Intra-articular infiltration with hyaluronic acid in the 6 months before inclusion; (3) Angular alterations higher than 15 degrees and unstable joint; (4) Systemic or local infectious and polyarticular diseases; (5) Undergoing oncological treatments or follow-ups; (6) Immunosuppressive treatment or systemic autoimmune diseases; (7) Poorly controlled hypertension or diabetes; (8) Allergy to any study drugs or excipients; (9) Patients on anticoagulants or anti-platelet therapy which could not be reversed temporarily for infiltrations; (10) Pregnancy; (11) Positive for syphilis, hepatitis B, hepatitis C or HIV; and (12) Incapable of understanding or fulfilling study questionnaires.

### 2.3. Randomization and Blinding

Patients were selected consecutively as they attended the Orthopedic Surgery and Traumatology Unit, meeting the inclusion criteria but none of the exclusion criteria. The study was blind to the patients and to the evaluators of the response. Each patient was assigned a code for the study and randomly allocated in a 1:1 ratio to one of two study groups using permuted blocks, a process carried out by the Clinical Pharmacology Unit. The result of this randomization was kept in that unit and was not accessible to researchers. Researchers were given opaque, sealed envelopes, each with a number on the outside corresponding to the order of entry into the trial (patient code). Inside each envelope was the indicated treatment. In order to keep the patients unaware of the treatment they were receiving, the same amount of blood was drawn from all patients prior to each infiltration, regardless of treatment group. Throughout the clinical trial, the patient was unaware of which group they were assigned to. When the patient arrived for treatment, the researcher responsible for preparing it opened the envelope to find out which treatment was to be administered. Similarly, to ensure that neither the patients nor the rest of the researchers knew which group they were in, the syringes used for the intraosseous application of PRGF or saline placebo were covered with stickers once they had been prepared. Furthermore, the researcher who prepared the injections was different from the one who administered them to the patients. The effectiveness of the blinding was not evaluated. The statistical analysis was carried out blindly.

### 2.4. Preparation of Plasma Rich in Growth Factors (PRGF)

PRGF-Endoret was prepared according to the Anitua et al. method [33]. In short, 72 mL of peripheral venous blood was drawn for the first round of treatment (combined intraosseous and intra-articular infiltrations), and 36 mL for the second and third rounds (only intra-articular infiltrations). The blood was collected in 9 mL tubes containing 400 μL of 3.8% sodium citrate as an anticoagulant (Endoret Traumatology kit, BTI Biotechnology Institute, S.L., Vitoria, Spain). The blood tubes were then centrifuged for eight minutes at 580× *g* in the PRGF-Endoret System centrifuge. The upper plasma volume was discarded, and the 2 mL plasma fraction located just above the buffy coat (F2 fraction) was collected without aspirating any leukocytes or erythrocytes. PRGF activation (F2 fraction) was performed just before infiltration by adding PRGF activator (10% *w*/*v* calcium chloride) in a ratio of 20 μL per milliliter of PRGF.

### 2.5. Interventions

The treatment plan comprises a series of three infiltrations administered at intervals of one to two weeks. The first infiltration was a combination of intraosseous PRGF or saline solution (depending on the study group) and intra-articular PRGF. The second and third injections consisted of only intra-articular PRGF for both groups. Prior to IO infiltration, patients were sedated with an infusion of propofol (1.5 mg/kg body weight) and fentanyl (75 μg). IO infiltration was performed under fluoroscopy. A volume of between 3 and 4 mL of PRGF or saline solution was injected into the tibial plateau, and an equivalent amount into the femoral condyle. For subsequent pain management, 50 mg of dexketoprofen was administered in 100 mL of normal saline solution. Finally, both study groups received two series of weekly 6–8 mL intra-articular PRGF, administered 1–2 weeks after the intraosseous infiltration.

### 2.6. Outcome Assessments

Patients were evaluated before treatment (baseline) and at 3, 6, and 12 months after the end of treatment using validated instruments: the Knee Injury and Osteoarthritis Outcome Score (KOOS) and the Western Ontario and McMaster Universities Osteoarthritis Index (WOMAC). Patients would not take any painkillers for 48 h before completing said questionnaires. The WOMAC questionnaire includes three domains: pain, stiffness, and physical function. A 5-point Likert scale was used for each item, and a score was obtained in each domain. These scores were then added up to achieve an aggregate score (WOMAC total) ranging from 0 to 96. A higher WOMAC score indicates more severe pain, stiffness and functional limitation [34,35]. On the other hand, the KOOS questionnaire is composed of five subscales which are measured separately: KOOS Pain, KOOS Symptoms, KOOS Activities of Daily Living, KOOS Sports/Recreation, and KOOS Quality of Life. For each item, a 5-point Likert scale was employed, and the resulting scores were converted to a 0–100 numeric scale, where 0 represented an extremely poor knee condition and 100 represented optimal knee health. In accordance with the instructions of Ross et al., an overall score was not computed [36]. The primary outcome was the KOOS scale measured at 6 months. Any adverse events reported during the clinical trial were also recorded.

### 2.7. Statistical Analysis

We conducted a priori power analysis to determine the necessary sample size for detecting differences between groups in the KOOS scale at 6 months, based on Sanchez et al.’s prior study [25] evaluating the efficacy of this treatment in KOA. A difference in equivalence of 10 points on the KOOS scale, with a standard deviation of 15, was considered at a statistical power of 80% and an alpha level of 0.05. It was calculated that a sample size of 37 subjects was required in each group. Assuming a 10% dropout rate throughout the study, the final sample size in each group was 43 patients.

Results were reported as the mean ± standard deviation and 95% CI of the mean. Descriptive data were presented as frequencies and percentages. All data values were tested for normality using the Shapiro–Wilk test. A repeated measures general linear model (GLM) with a Bonferroni test for multiple comparisons was performed to assess differences at various follow-up times within each group. For each variable (e.g., KOOS symptoms), the intrasubject factor ‘time’ (with four levels) and the intersubject factor ‘group’ (with two levels: control and PRGF) were analyzed. Mauchly’s test was used to test the sphericity. Pillai’s Trace was used in the absence of sphericity. Comparative analysis between treatment groups was based on either a two-tailed Student’s *t*-test (univariate) or a non-parametric Mann–Whitney U-test, depending on whether the values followed a normal distribution. The analysis was conducted per-protocol. No imputation method was used for missing data. On the other hand, the change in joint pain from baseline was assessed using the KOOS pain subscale. Success rates were calculated based on a reduction in pain score of at least 9.3 points from baseline, which is considered the minimum clinically important improvement (MCII) [37]. Pearson’s chi-square test was performed to compare the percentage of patients who exceeded the MCII. The relationship between demographics and clinical outcomes was analyzed with the Spearman rank correlation test.

The box-and-whisker plots were generated following Tukey-style: the mean has the symbol +, the horizontal line inside of the boxes represents the median, the boxes show the IQR, while the whiskers indicate the 25th percentile-1.5× the IQR and the 75th percentile-1.5× the IQR. The round points outside the whisker range indicate the outliers [38]. Differences were considered statistically significant at *p* < 0.05. Statistical analyses were performed using SPSS software (version 15.0, IBM, Chicago, IL, USA). Box-and-whisker plots were created with GraphPad Prism (version 10.6, GraphPad Software, San Diego, CA, USA).

## 3. Results

### 3.1. Demographic Characteristics

**Figure 1:**
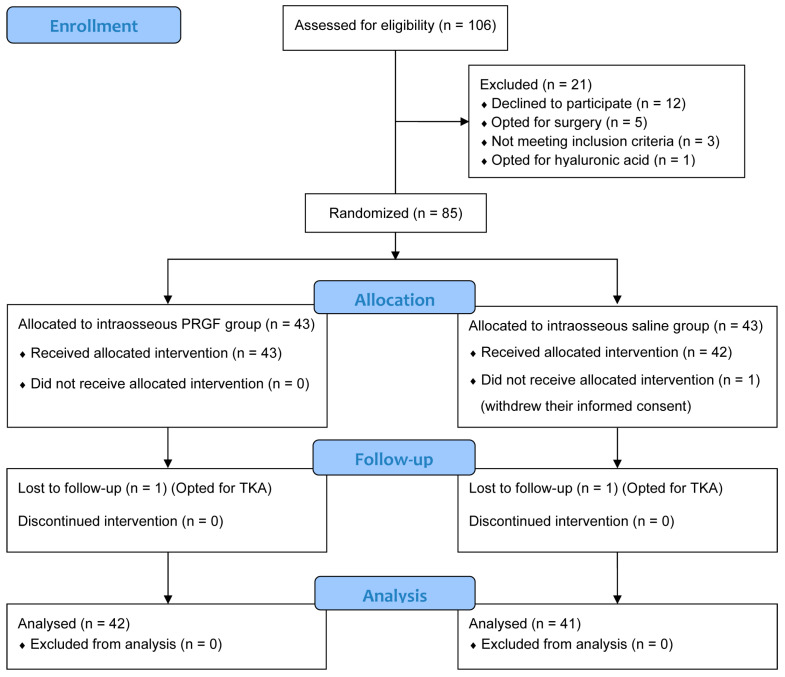
CONSORT (Consolidated Standards of Reporting Trials) 2025 flow diagram of the randomized controlled trial (RCT). PRGF, Plasma rich in growth factors; TKA, Total knee arthroplasty.

Between October 2017 and March 2022, 106 patients were assessed for study eligibility. Of these, 21 were excluded and 86 patients were finally randomized and allocated to the intraosseous PRGF group (n = 43) or intraosseous saline group (n = 43), but one patient in the saline group withdrew their informed consent and did not receive the treatment (Figure 1). Two patients dropped out of the study, one from each group, and chose to undergo total knee replacement instead. Finally, 42 patients were analyzed in the intraosseous PRGF group while 41 patients were in the intraosseous saline group. Both groups were homogeneous at baseline for all the parameters, without any statistically significant differences in terms of sex, age, body mass index, laterality, OA grade or baseline scores (Table 1).

### 3.2. KOOS Outcome Assessment

**Figure 2:**
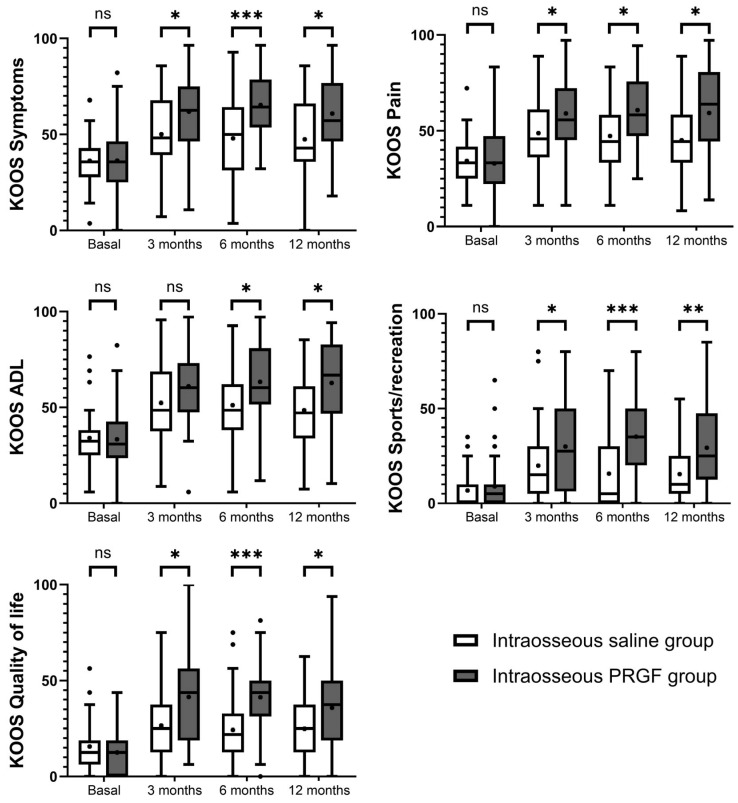
Graphical representation of the results of the KOOS questionnaires. Higher scores indicate less pain and greater functionality. ns, non-significant, * indicates *p* < 0.05, ** indicates *p* < 0.01, and *** indicates *p* < 0.001.

Figure 2 illustrates the follow-up for this variable. Regarding the intra-group analysis, both the intraosseous PRGF group and the intraosseous saline group showed statistically significant improvements from baseline in all five KOOS domains at the 3, 6, and 12-month follow-up points (Table 2). For the intraosseous PRGF group, the improvements in Symptoms, Pain, Activities of Daily Living, Sports/recreation, and Quality of Life were highly significant (*p* < 0.001) at all time-points compared to their baseline values. The intraosseous saline group also experienced significant improvements from baseline at all follow-ups across all subscales, with *p* values ranging from 0.047 to <0.001. In the intergroup comparison (Table 2), no statistically significant differences were observed between the groups at baseline for any of the KOOS subscales. However, at all subsequent follow-up points, the IO PRGF group showed significantly greater improvement than the IO saline group in nearly all domains. Specifically, for the KOOS Symptoms, Pain, Sports/recreation, and Quality of Life subscales, the intraosseous PRGF group’s scores had significantly greater improvement at 3, 6, and 12 months (Table 2). For the KOOS Activities of Daily Living domain, the intraosseous PRGF group also demonstrated significantly better results at 6 months (*p* = 0.007) and 12 months (*p* = 0.002), though the difference at 3 months did not reach statistical significance (*p* = 0.050).

### 3.3. WOMAC Outcome Assessment

**Figure 3:**
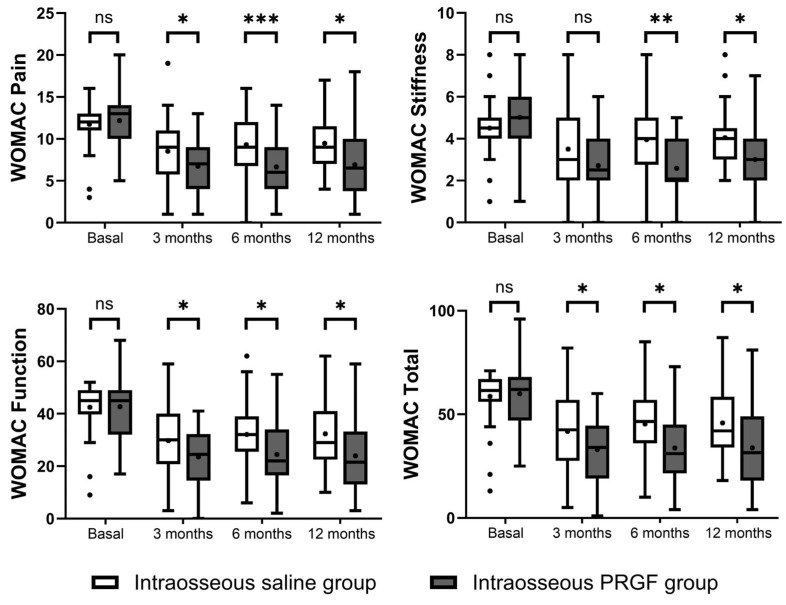
Representation of the results of the WOMAC questionnaires. Higher scores indicate greater pain and poorer functionality. ns, non-significant, * indicates *p* < 0.05, ** indicates *p* < 0.01, and *** indicates *p* < 0.001.

The intragroup analysis based on the WOMAC score indicated significant improvements in both treatment groups (Figure 3). The intraosseous PRGF group demonstrated highly significant improvements from baseline across all WOMAC domains (Pain, Stiffness, and Function) and in WOMAC Total at the 3, 6, and 12-month follow-ups (*p* < 0.001 for all variables and follow-up times) (Table 3). The intraosseous saline group also showed significant improvements from baseline in the Pain, Function, and Total scores at all time-points. However, for the WOMAC Stiffness domain, the saline group showed a significant improvement only after 3 months (*p* = 0.018), with no significant changes from baseline observed at 6 (*p* = 0.442) and 12 months (*p* = 0.431).

At baseline, there were no statistically significant differences between the two groups in any of the WOMAC domains. In the intergroup comparison at follow-up, the intraosseous PRGF group consistently showed statistically superior outcomes compared to the intraosseous saline group (Figure 3). The improvement in the intraosseous PRGF group was significantly greater for WOMAC Pain, Function, and Total scores at all follow-up points (3, 6, and 12 months). For the WOMAC Stiffness domain, the difference between the groups was not significant at 3 months (*p* = 0.077), but the intraosseous PRGF group showed significantly greater improvement at the 6-month (*p* = 0.001) and 12-month (*p* = 0.006) evaluations (Table 3).

### 3.4. Effect of PRGF on the MCII—Pain

**Figure 4:**
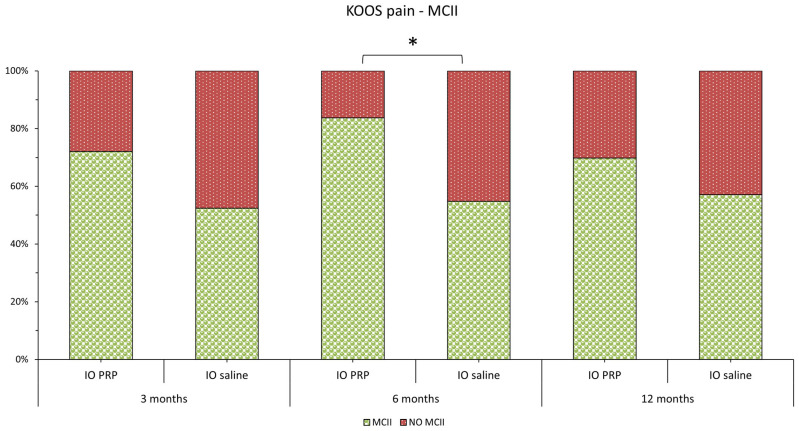
Graphical representation of the percentage of patients in each study group who achieve MCII for the KOOS Pain scale (The green bars show the percentage of patients who achieve MCII, while the red bars show the percentage who do not). KOOS, Knee Injury and Osteoarthritis Outcome Score; MCII, Minimum clinically important improvement; IO, intraosseous; PRGF, Plasma rich in growth factors. * indicates *p* < 0.05.

Most patients in the study were able to achieve MCII (Figure 4). The percentage of patients in the intraosseous PRGF group who achieved MCII was consistently higher than that in the intraosseous saline group, although this difference was only statistically significant after 6 months of follow-up (*p* = 0.0606 after 3 months, *p* = 0.0038 after 6 months, and *p* = 0.2267 after 12 months).

### 3.5. Analysis of Correlation

Correlations were performed to establish whether any demographic or diagnostic factors (such as age, sex, weight, height, BMI, or degree of osteoarthritis) could influence the clinical outcome. No significant correlation was observed in the intraosseous saline group (*p* > 0.05). In contrast, several associations were observed in the intraosseous PRGF group. The main correlation was found to be between the age variable and the clinical outcome. Thus, a correlation was observed between age and KOOS Pain at all follow-up times (R = 0.420, *p*= 0.039; R = 0.359, *p* = 0.047; R = 0.311, *p* = 0.047; at 3, 6 and 12 months, respectively), with KOOS ADL also at all follow-up times (R = 0.427, *p*= 0.029; R = 0.366, *p* = 0.037; R = 0.292, *p* = 0.049, at 3, 6 and 12 months, respectively), and with KOOS Symptoms (R = 0.422, *p* = 0.006), KOOS Sports (R = 0.398, *p* = 0.022) and KOOS QoL (R = 0.399, *p* = 0.046) at the three-month follow-up. On the WOMAC scale, a correlation was observed between age and clinical outcome with WOMAC Pain (R = −0.350, *p* = 0.027) and WOMAC Total (R = −0.364, *p* = 0.049) at three months and with WOMAC Stiffness at 12 months (R = −0.353, *p* = 0.029). Similarly, and only for the intraosseous PRGF group, a correlation was found between weight (and not with BMI) and all WOMAC scale domains at 6 months: WOMAC Pain (R = 0.320, *p* = 0.036), WOMAC Stiffness (R = 0.301, *p* = 0.024), WOMAC Function (R = 0.352, *p* = 0.007), and WOMAC Total (R = 0.368, *p* = 0.008). At 12 months, a correlation was also found in the PRGF group between weight and the KOOS Sports (R = −0.403, *p* = 0.031) and WOMAC Stiffness (R = 0.312, *p* = 0.048).

### 3.6. Adverse Events

No patients experienced any serious adverse reactions. Only 15 patients, 7 in the PRGF group and 8 in the saline one, reported pain and inflammation in the first 72 h, which were spontaneously resolved. There were no differences between the two treatment groups.

## 4. Discussion

This study shows that both intraosseous PRGF and saline solution infiltrations, in combination with intra-articular PRGF infiltrations, significantly improved KOOS and WOMAC scales 3 months after the end of treatment and maintained these ameliorations up to 1 year follow-up with respect to patient baseline conditions. However, the intra-osseous PRGF group presented significantly better clinical outcomes pertaining to pain reduction and functional improvement as compared to the intraosseous saline group. To the best of our knowledge, no previous study has analyzed the effect of IO PRGF infiltrations as compared to IO saline placebo, both of which were followed by active treatment with IA PRGF.

Our data is in agreement with other studies published on patients with severe KOA applying similar treatment protocols involving PRGF [25,26,28]. Interestingly, the average values of the MCII in the KOOS pain subscore of 52.4% at 3 months, 54.8% at 6 months, and 57.1% at 12 months for the patients who underwent the saline control treatment were close to those published by Sanchez et al. in 2022 [27] (46% at 6 months and 56% at 15 months) for patients with severe KOA treated with only 3 intra-articular injections of PRGF and both were inferior to the average in KOOS pain subscore (MCII) of 75% between 6 and 24 months of follow-up for patients with severe KOA treated with a combination of IO and IA PRGF infiltrations published by Rios Luna et al. [28]. Importantly, in our study, the group treated with IO PRGF showed a superior improvement of MCII in the KOOS pain subscale of 19.7% at 3 months, 28.9% at 6 months and 12.7% at 12 months as compared with the IO saline control values, which parallel those presented by Sanchez et al. [25,27] of 13% and 12% at 6 and 15 months, respectively, when comparing the combination of IO and IA with only IA PRGF administration. Overall, our results strongly suggest that tackling the subchondral bone with PRGF instead of saline solution exerts a superior biological and clinical summatory effect, as reflected by the long-term pain reduction and amelioration of joint function in our study group [25,27,28].

Several factors operating synergistically might account for the clinical improvement, including the beneficial effect of bioactive molecules conveyed by the injected PRGF together with its dilution effect on the pro-inflammatory tissue microenvironment thereby reducing the concentration of pro-inflammatory cytokines which sensitize myriad nociceptor endings at the bone trabecular injured, and finally, the mechanical core decompression effect as a consequence of inserting the trocar in the subchondral bone [39]. This latter effect, also shared by saline infiltration, is thought to be due to an increase in venous drainage and reduction in pressure of the subchondral vascular system which improves the inflow of nutrients [39]. Moreover, it has been suggested that saline infiltration exerts an anti-inflammatory effect by increasing the osmolarity of the injured area, an effect independent of the type of solute, which reduces the activation of neutrophils [40]. Overall, our study somehow corroborates the above, since saline infiltrations also improved the clinical outcomes, although to a lesser extent than PRGF, a fact likely associated with the absence of bioactive molecules as is the case with saline injections.

The data thus points to PRGF as the active agent in causing middle and long-term antialgic, anti-inflammatory and trophic effects on the joint. PRGF operates as a dynamic, nonlinear, combinatorial, synergistic and multidirectional biological system throughout several biomolecules conveyed gradually by the PRGF fibrin matrix (TGF-β, HGF and IGF-1 among others) in a context-dependent manner [3,12,18]. The exact mechanisms by which this therapeutic strategy exerts anti-inflammatory, analgesic, and trophic effects have not yet been fully determined and are based on preclinical research that must be validated through clinical trials. One robust candidate is the inhibitory effect of HGF, PDGF, and IGF-1 conveyed by PRGF on the NF-kB signaling pathway of stressed and injured synovial joint cells mediating the inflammatory response [41,42,43,44], thus contributing to the anti-inflammatory action. Moreover, it has been reported that IO PRGF, through the concurrent presence of HGF, TGFβ1 and IGF-1, might exert a senolytic activity on subchondral mesenchymal stromal cells (MSCs) by restoring the osteogenic microenvironment and the TGF-β homeostasis [45,46,47,48], as well as anti-inflammatory and anti-fibrotic effects on the synovial membrane through fibroblast-like synoviocytes [49]. In this line, the dwindling of the pain-generating pathological fibroneurovascular subchondral tissue in KOA and the restoration of the TGFβ1 and HGF balance could reduce the synthesis of profibrotic mediators such as NGF and VEGF, thereby contributing to attenuating or eliminating pain [3,9,10,12,50,51]. On the other hand, several studies have reported that this treatment boosts the proliferation [52] and increases the stress-resistance capacity of bone marrow MSCs [53] which, together with the senolytic activity on subchondral MSCs, might well contribute to attenuating or eliminating pain [54,55,56]. PRGFs are an important source of lipoxin A4 (LXA 4), an endogenous pro-resolving lipid mediator of inflammation derived from arachidonic acid, and positively contribute to the resolution of inflammation mediated by some types of PRGFs [57,58,59]. Significantly, PRGFs, in addition to containing a significant concentration of endogenous endocannabinoids, also stimulate endogenous endocannabinoid synthesis, acting as ligands for cannabinoid receptors 1 (CB1) and 2 (CB2) of chondrocytes, synovial cells and bone cells, and decrease the excitability of nociceptors, thus contributing to analgesic action [60,61,62]. The aforementioned mechanisms are not mutually exclusive and might well operate synergistically in order to reduce inflammation and pain at the synovial membrane and subchondral bone [12], both well-vascularized and innervated tissues where the polarization effects of PRGF on M1 to M2 [63,64] might lessen the detrimental influence of the systemic low-grade inflammation [65,66,67]. An RCT demonstrated a molecular correlation with the clinical outcome [68], showing that patients treated with intra-articular or intraosseous PRP injections had lower inflammation marker levels than those given a placebo. Furthermore, patients who received intraosseous injections exhibited a reduction in cartilage degradation markers compared to those who received only intra-articular injections [68].

Correlation analysis of patient demographic factors and clinical outcomes revealed significant correlations only in the intraosseous PRGF group. In this group, older age was found to be associated with higher scores in several KOOS domains and lower scores in several WOMAC domains. Given the antagonistic nature of these scales, this implies that older age was associated with better joint health outcomes (less pain and greater functionality). Similarly, a correlation was found between several clinical variables and high weight, which was identified as a poor prognostic factor. In a large number of cases, the correlation found (R between 0.30 and 0.50) can be classified as moderate [69]. Unlike Sanchez et al. [37], we found no correlation between patient gender and clinical outcome. However, the article [37] only performs intra-articular injections, unlike our research, which involves both intra-articular and intraosseous PRGF injections. Saraf et al. [70] also found no correlation, but like Sánchez et al., the infiltrations were solely intra-articular. A more in-depth analysis is required to investigate the causes of these correlations, since advanced age and a higher BMI are two of the main factors contributing to both the progression of KOA and the probability of undergoing surgery [71].

Several systematic reviews and meta-analyses have examined the effectiveness of PRP and PRGF for treating KOA. Bagheri et al. [72], Xu et al. [73] and Li et al. [74] compared the efficacy of PRP versus hyaluronic acid (HA), demonstrating that PRP is superior to viscosupplementation after 12 months. In 2023, Xiong et al. [75] demonstrated that PRP is more effective than HA or a placebo, attributing better analgesic properties to PRP without leukocytes. Similarly, Migliorini et al. [76] conducted a meta-analysis but only included PRGF. They found that PRGF could be associated with more favorable clinical outcomes than HA on some WOMAC scales, though not all. However, this review did not include all PRGF studies, and not all of the studies included actually involved PRGF. Unfortunately, none of these systematic reviews evaluate the efficacy of PRP through intraosseous infiltration; they all focus on intra-articular infiltration. However, although systematic reviews analyzing the intraosseous route for treating KOA [77,78,79] support this approach, they include few studies, many of which are not RCTs which limits their conclusions. Real-world evidence is another body of evidence supporting the use of intraosseous PRP, with studies [27,80] on more than 300 patients demonstrating its ability to delay the need for surgery.

This clinical trial, however, presents several limitations that warrant discussion. It has not been possible to characterize the PRGF applied to each patient. Nevertheless, the reliability of the processing and manufacturing of PRGF has been demonstrated extensively in multiple studies, yielding a PRGF generally classified as 24-00-11 according to the latest coding system [81,82]. Furthermore, this study had already been initiated when the MIBO recommendations were published [83]. As to the design of this study, a third control group supplying the subchondral bone with no saline placebo would have helped discern just how far the mechanical and core decompression effect went to improving patient symptoms. Moreover, in order to enrich the study, we should have included more patients in the second participating hospital center. The fact that most enrolled patients underwent bilateral treatment reflects the real pragmatic symmetrical impact of KOA [84] and we considered it important for it to be included. In order to make the population more homogeneous, our RCT has limited both age (40–75 years) and BMI (<35). However, it is well-established that both age [85,86] and BMI [87] are risk factors for the development of KOA. Therefore, in order to draw valid conclusions for a wider population, it would be advisable to conduct studies in older populations and in people with a higher BMI. Finally, in order to assess whether the combination of IO and IA PRGF infiltrations exerts structural modifications on joint tissues, it would have been advisable to perform control imaging studies such as MRI to examine the impact of this treatment as a structure-modifying strategy. This type of objective analysis would add value to the RCT, as we have only analyzed subjective variables (KOOS and WOMAC).

## 5. Conclusions

In conclusion, combining intraosseous and intra-articular injections of PRGF in patients with severe KOA has proven to have a summatory effect on outcome, with direct antialgic effects and joint function improvement, as compared to intraosseous saline solution and intra-articular PRGF infiltrations.

## Figures and Tables

**Table 1:** Characteristics of patients included in the two treatment groups ^a^.

	IO PRGF (n = 42)	IO Saline (n = 41)	*p* Value
Sex			0.918
Male; n (%)	19 (45.2)	20 (48.8)	
Female; n (%)	23 (54.8)	21 (51.2)	
Age (years)	60.1 ± 7.5	59.5 ± 7.4	0.691
Weight (kg)	80.6 ± 11.8	76.2 ± 10.8	0.084
Height (m)	1.69 ± 0.09	1.66 ± 0.10	0.157
Body mass index (kg/m^2^)	28.1 ± 2.6	27.6 ± 2.5	0.157
Affected side; n (%)			0.435
Left	12 (28.6)	7 (17.1)	
Right	12 (28.6)	15 (36.6)	
Bilateral	18 (42.9)	19 (46.3)	
Kellgren–Lawrence OA grade; n (%)			0.943
Grade III	30 (71.4)	28 (68.3)	
Grade IV	12 (28.6)	13 (31.7)	
KOOS scores			
KOOS Symptoms	35.4 ± 15.6	36.9 ± 14.6	0.983
KOOS Pain	31.8 ± 15.3	35.2 ± 13.1	0.690
KOOS ADL	32.7 ± 15.7	34.2 ± 13.7	0.745
KOOS Sports/recreation	7.5 ± 12.9	7.3 ± 10.4	0.542
KOOS Quality of life	12.0 ± 12.7	15.1 ± 11.6	0.123
WOMAC scores			
WOMAC Pain	12.2 ± 3.6	11.9 ± 2.7	0.793
WOMAC Stiffness	5.0 ± 1.6	4.6 ± 1.4	0.147
WOMAC Function	43.1 ± 11.4	43.2 ± 8.4	0.892
WOMAC Total	60.3 ± 15.7	59.7 ± 11.4	0.993

^a^ Data are expressed as mean ± SD or n (%). KOOS, Knee Injury and Osteoarthritis Outcome Score; IO, intraosseous; OA, Osteoarthritis; PRGF, Plasma rich in growth factors; ADL, Activities of Daily Living. WOMAC, Western Ontario and McMaster Universities Osteoarthritis Index. Percentages might not always add up to exactly 100% as a result of rounding.

**Table 2:** KOOS outcome assessment at baseline 3, 6 and 12 months ^a^.

	IO PRGF (n = 42)			IO Saline (n = 41)			*p* Value Intergroup ^c^
Outcome Variable	Mean ± SD	95% CI of Mean	*p* Value	Mean ± SD	95% CI of Mean	*p* Value	
KOOS Symptoms							
Basal	35.4 ± 15.6	31.11–41.65	-	36.9 ± 14.6	31.88–40.74	-	0.983
3 months	61.3 ± 19.1	55.70–68.04	**<0.001** ^b^	50.1 ± 21.5	43.31–56.88	**0.001** ^b^	**0.011**
6 months	64.8 ± 16.6	60.38–70.34	**<0.001** ^b^	48.7 ± 22.6	41.09–54.83	**0.007** ^b^	**0.000**
12 months	61.8 ± 18.9	54.79–66.97	**<0.001** ^b^	48.1 ± 21.0	40.98–53.99	**0.007** ^b^	**0.003**
3 months–6 months			0.594			1	
3 months–12 months			1			1	
6 months–12 months			1			1	
KOOS Pain							
basal	31.8 ± 15.3	27.68–38.20	-	35.2 ± 13.1	30.18–38.34	-	0.690
3 months	57.5 ± 19.8	52.47–65.57	**<0.001** ^b^	49.0 ± 18.6	42.93–54.71	**<0.001** ^b^	**0.022**
6 months	60.3 ± 20.6	54.56–66.98	**<0.001** ^b^	48.1 ± 19.6	41.31–53.28	**<0.001** ^b^	**0.002**
12 months	59.8 ± 20.9	52.54–65.99	**<0.001** ^b^	46.0 ± 18.5	39.18–50.91	**0.005** ^b^	**0.002**
3 months–6 months			1			1	
3 months–12 months			1			1	
6 months–12 months			1			1	
KOOS ADL							
basal	32.7 ± 15.7	28.04–38.64	-	34.2 ± 13.7	29.92–38.21	-	0.745
3 months	60.6 ± 19.4	54.85–67.28	**<0.001** ^b^	52.8 ± 19.7	46.11–58.67	**<0.001** ^b^	0.050
6 months	62.8 ± 20.1	57.43–69.40	**<0.001** ^b^	51.9 ± 21.4	44.63–57.82	**<0.001** ^b^	**0.007**
12 months	64.0 ± 19.2	56.29–69.22	**<0.001** ^b^	49.7 ± 19.1	42.37–54.63	**<0.001** ^b^	**0.002**
3 months–6 months			1			1	
3 months–12 months			1			1	
6 months–12 months			1			1	
KOOS Sports/recreation							
basal	7.5 ± 12.9	4.52–13.39	-	7.3 ± 10.4	3.62–9.96	-	0.542
3 months	28.5 ± 23.1	22.44–37.40	**<0.001** ^b^	20.4 ± 20.5	13.33–26.42	**0.001** ^b^	**0.048**
6 months	33.3 ± 20.1	28.77–41.69	**<0.001** ^b^	16.7 ± 19.5	9.623–21.57	**0.019** ^b^	**0.000**
12 months	29.7 ± 23.4	22.04–36.58	**<0.001** ^b^	16.2 ± 16.2	10.27–20.46	**0.016** ^b^	**0.004**
3 months–6 months			0.627			0.604	
3 months–12 months			1			0.815	
6 months–12 months			1			1	
KOOS Quality of life							
basal	12.0 ± 12.7	8.44–16.60	-	15.1 ± 11.6	11.85–19.46	-	0.123
3 months	39.2 ± 20.2	34.46–48.42	**<0.001** ^b^	26.3 ± 18.7	20.66–32.49	**0.010** ^b^	**0.002**
6 months	40.4 ± 17.8	35.69–46.92	**<0.001** ^b^	24.7 ± 18.8	18.60–29.96	**0.047** ^b^	**0.000**
12 months	36.0 ± 19.7	29.70–41.99	**<0.001** ^b^	24.5 ± 17.3	19.51–30.22	**0.019** ^b^	**0.008**
3 months–6 months			1			1	
3 months–12 months			1			1	
6 months–12 months			1			1	

^a^ Values are expressed as mean ± SD and 95% CI of mean. ^b^ Intragroup comparison with basal values, ^c^ Comparison between the 2 groups at each follow-up point. CI, Confidence Interval; KOOS, Knee Injury and Osteoarthritis Outcome Score; IO, intraosseous; PRGF, Plasma rich in growth factors; ADL, Activities of Daily Living. Statistically significant differences (*p* < 0.05) are in **boldface.**

**Table 3:** WOMAC outcome assessment at baseline 3, 6 and 12 months ^a^.

	IO PRGF (n = 42)			IO Saline (n = 41)			*p* Value Intergroup ^c^
Outcome Variable	Mean ± SD	95% CI of Mean	*p* Value	Mean ± SD	95% CI of Mean	*p* Value	
WOMAC Pain							
Basal	12.2 ± 3.6	11.03–13.34	-	11.9 ± 2.7	10.85–12.63	-	0.793
3 months	6.8 ± 3.3	5.64–7.84	**<0.001** ^b^	8.5 ± 3.8	7.29–9.77	**<0.001** ^b^	**0.032**
6 months	6.9 ± 3.5	5.54–7.78	**<0.001** ^b^	9.2 ± 3.6	8.15–10.47	**0.001** ^b^	**0.001**
12 months	6.9 ± 3.6	5.65–8.16	**<0.001** ^b^	9.5 ± 3.3	8.44–10.49	**0.005** ^b^	**0.002**
3 months–6 months			1			1	
3 months–12 months			1			0.916	
6 months–12 months			1			1	
WOMAC Stiffness							
basal	5.0 ± 1.6	4.51–5.53	-	4.6 ± 1.4	4.04–4.96	-	0.147
3 months	2.7 ± 1.6	2.19–3.23	**<0.001** ^b^	3.5 ± 1.9	2.86–4.14	**<0.018** ^b^	0.077
6 months	2.5 ± 1.4	2.16–3.01	**<0.001** ^b^	3.9 ± 2.1	3.31–4.59	0.442 ^b^	**0.001**
12 months	3.0 ± 1.7	2.49–3.52	**<0.001** ^b^	4.0 ± 1.4	3.58–4.52	0.431 ^b^	**0.006**
3 months–6 months			1			0.899	
3 months–12 months			1			0.403	
6 months–12 months			0.664			1	
WOMAC Function							
basal	43.1 ± 11.4	39.16–46.29	-	43.2 ± 8.4	39.68–45.32	-	0.892
3 months	23.9 ± 10.5	20.08–27.02	**<0.001** ^b^	29.8 ± 12.7	25.63–33.95	**<0.001** ^b^	**0.022**
6 months	25.1 ± 12.3	20.70–28.33	**<0.001** ^b^	31.6 ± 13.4	27.83–36.36	**<0.001** ^b^	**0.009**
12 months	23.6 ± 12.8	19.71–28.20	**<0.001** ^b^	32.2 ± 12.2	28.49–36.19	**<0.001** ^b^	**0.004**
3 months–6 months			1			1	
3 months–12 months			1			1	
6 months–12 months			1			1	
WOMAC Total							
basal	60.3 ± 15.7	54.96–64.90	-	59.7 ± 11.4	54.86–62.62	-	0.993
3 months	33.4 ± 14.6	28.19–37.81	**<0.001** ^b^	41.8 ± 17.8	35.96–47.67	**<0.001** ^b^	**0.021**
6 months	34.6 ± 16.5	28.62–38.90	**<0.001** ^b^	44.7 ± 18.4	39.51–51.21	**<0.001** ^b^	**0.004**
12 months	33.5 ± 17.5	28.03–39.68	**<0.001** ^b^	45.7 ± 16.6	40.65–51.06	**<0.001** ^b^	**0.003**
3 months–6 months			1			1	
3 months–12 months			1			0.930	
6 months–12 months			1			1	

^a^ Values are expressed as mean ± SD and 95% CI of mean. ^b^ Intragroup comparison with basal values, ^c^ Comparison between the 2 groups at each follow-up point. CI, Confidence Interval; WOMAC, Western Ontario and McMaster Universities Osteoarthritis Index; IO, intraosseous; PRGF, Plasma rich in growth factors. Statistically significant differences (*p* < 0.05) are in **boldface**.

## Data Availability

All the data obtained to support the findings of this study are available from the corresponding authors upon reasonable request.

## Supplementary Materials

The following supporting information can be downloaded at: https://www.mdpi.com/article/10.3390/jcm14228075/s1, Table S1: CONSORT 2025 statement checklist.

## Abbreviations

The following abbreviations are used in this manuscript:

ADL	Activities of Daily Living
CI	Confidence Interval
CONSORT	Consolidated Standards of Reporting Trials
GLM	General linear model
HA	Hyaluronic acid
IA	Intraarticular
IO	Intraosseous
KOA	Knee osteoarthritis
KOOS	Knee Injury and Osteoarthritis Outcome Score
MCII	Minimum clinically important improvement
MSCs	Mesenchymal stromal cells
NS	Non-significant
PRGF	Plasma rich in growth factors
PRP	Platelet-rich plasma
RCT	Randomized Controlled Trial
QoL	Quality of life
WOMAC	Western Ontario and McMaster Universities Osteoarthritis Index
